# Conceptual Design of Composite Sandwich Structure Submarine Radome

**DOI:** 10.3390/ma12121966

**Published:** 2019-06-18

**Authors:** Hafiz Muhammad Waqas, Dongyan Shi, Muhammad Imran, Sohaib Z. Khan, Lili Tong, Fazal e Ahad, Asad A. Zaidi, Javid Iqbal, Waqas Ahmed

**Affiliations:** 1 College of Mechanical and Electrical Engineering, Harbin Engineering University, Harbin 150001, China; hafizwaqas@hrbeu.edu.cn (H.M.W.); mimran@hrbeu.edu.cn (M.I.); ahad_khattak@hotmail.com (F.e.A.); javid784@hrbeu.edu.cn (J.I.); waqas@hrbeu.edu.cn (W.A.); 2 Department of Mechanical Engineering, Faculty of Engineering, Islamic University of Madinah, Madinah 41411, Saudi Arabia; szkhan@iu.edu.sa; 3 Department of Engineering Sciences, PN Engineering College, National University of Sciences and Technology, Karachi 75350, Pakistan; asadali@pnec.nust.edu.pk; 4 College of Aerospace and Civil Engineering, Harbin Engineering University, Harbin 150001, China; tonglili@hrbeu.edu.cn

**Keywords:** submarine radome, sandwich core, composite failure criteria, buckling strength

## Abstract

Radomes are usually constructed from sandwich structures made of materials which usually have a low dielectric constant so that they do not interfere with electromagnetic waves. Performance of the antenna is increased by the appropriate assortment of materials enabling it to survive under marine applications, and it depends on composite strength-to-weight ratio, stiffness, and resistance to corrosion. The design of a sandwich core submarine radome greatly depends on the material system, number of layers, orientation angles, and thickness of the core material. In this paper, a conceptual design study for a sandwich core submarine radome is carried out with the help of finite element analysis (FEA) using two unidirectional composite materials—glass fiber reinforced polymer (GFRP) and carbon fiber reinforced polymer (CFRP)—as a skin material and six different core materials. Conceptual designs are obtained based on constraints on the composite materials’ failure, buckling, and strength. The thickness of the core is reduced under constraints on material and buckling strength. Finite element analysis software ANSYS WORKBENCH is used to carry out all the simulations.

## 1. Introduction

Due to the properties of high strength, light weight, flexibility of design, and excellent corrosion resistance, fiber reinforced polymers (FRPs) have been widely applied as strengthening and structural materials in marine, military, chemical, and civil infrastructure [1,2,3]. Recent engineering structures, particularly in the fields of wind turbines [4], aerospace [5], highway bridges [6], and ocean environments [7], have progressively emphasized important practical requirements that are hard to implement for traditional steel, concrete, and other traditional materials. Therefore, research on FRPs with multifunctionality, high performance, and adaptive structure systems is imperative.

A sandwich structure is a layered composite material constructed by attaching two rigid, thin skins to a thick, lightweight core [8]. Their explicit properties, such as high strength-to-weight ratio and high stiffness-to-weight ratio for bending, are the most important benefits of these configurations. They are also of significant interest for aeronautical and naval structures due to their high corrosion resistance [9,10].

Radio frequency (RF) systems are used in submarines for communications with the help of radar antenna. Radomes are used to protect the radar antenna and are placed over the antenna in a dome-shaped structure, thus being called ‘radomes’ [11]—the combination of the two words ‘radar’ and ‘dome’. The basic function of the radome is to protect the radar antenna from weather conditions, dust, and harmful gases. While selecting the radome, it must be assured that there will be minimal impact on electromagnetic waves and electrical properties of the radar antenna. Ideally, the radome is electrically undetectable. How well the radome functions depends on composition of its material and matching its configuration to the particular radio frequency range [12].

There are three different types of radomes; i.e., naval, ground-based, and airborne. Generally, an assortment of materials has been utilized for building radomes. Current naval and ground-based radomes are produced utilizing composite materials; for example, quartz, fiberglass, and aramid filaments held together with epoxy, polyester, and different resins [13]. Honeycomb and foam centers are frequently included among internal and external ‘skins’ of the radome to work as a low-dielectric-constant insertion material giving rigidity and strength. However, for underwater applications, the pressure is too high for the larger depths, so in this article, we have introduced different wood cores with low dielectric constant and higher strength than foam cores.

There are many different configurations of radomes at present, and are used to minimize the different parameters i.e., RF reflections, half wave, and electrically thin [14]. The best design for a specific application relies upon the mechanical prerequisites and operating frequency. Electrically thin radomes with wavelengths less than 0.1 [15] normally deliver good RF performance. This is on the grounds that signal reflections at the free-space/dielectric limit are counterbalanced by out-of-stage reflections from the dielectric/free-space limit on the opposite side of the dielectric material. Tragically, electrically slender radomes give almost no thermal protection and are not appropriate for areas with wide temperature limits and a necessity for controlled temperatures.

An alternative radome methodology that functions admirably is a configuration dependent on the half-wavelength-thick compact laminate. It is similar to the electrically thin arrangement in light of the fact that the reflections counteract. The wave ventures 180° through the cover, is reflected with a stage move of 180°, and voyages another 180° on the arrival trek to accomplish the net 180° stage move required for dropping.

There are two main types of radome configuration and designs, i.e., A-sandwich and C-sandwich. The A-sandwich radome design is comprised of a low-dielectric-constant foam or honeycomb center sandwiched between two slim laminates. Its maneuver is similar to that of the half-wavelength-thick compact laminate. In any case, it is 0.25 wavelengths thick in light of the fact that the reflection coefficients from the skins have a similar phase and amplitude. The round excursion for the reflection from the second skin is 0.5 wavelengths. The reflections with 180 degrees out of phase are cancelled.

A C-sandwich design is comprised of two foam core layers and three layers of skin. The thickness of each foam layer, and potentially the skins, can be tuned for ideal RF execution in the bands of interest. This can prompt numerous potential development combinations that can give great RF execution and high mechanical quality and strength. C-sandwich developments give preferred execution over A-sandwich radomes; be that as it may, the additional unpredictability increases material and work costs.

Electrically thin dielectric material is used to make inflatable radomes. Very low loss is achieved over wide-ranging bandwidths due to its capability of being electrically thin. The compromise for superior performance, in any case, is that they require a steady supply of air. Inflatable radomes must be upheld by an internally produced pneumatic force which is provided via air compressors or air blowers. So as to keep up satisfactory air pressure, inflatable radomes must be prepared with sealed areas at all entryways and a backup power supply to work the blowers consistently and under every single natural condition. There is the possibility for the radome to collapse and deflate if there is any interruption in power or the membrane suffers any damage. Maintenance and functional costs for this sort of radome as a rule, surpass, those of all other radome types.

Laminated fiber-strengthened composite materials and their sandwich structures are presently utilized all through the marine business. Composites are omnipresent in delight pontoon and racing yacht development; are generally used in the development of quick ships, submarines, and maritime and coastguard watch specialties; angling and work water crafts; and furthermore in the seaward oil and gas industry. Composite materials produced using E-glass filaments and epoxy resin have turned out to be very well-known as radome materials because of their extraordinary transparency to microwaves and excellent mechanical properties. The expanding use of these materials for submerged applications presents difficulty to the originator in choosing the right construction process and shape of a radome because of the perplexing nature of their loading conditions and structure. They also provide resistance to the corrosion, which is the major cause of magnetic and electronic signatures.

The mechanical properties of composite materials normally rely upon their structure and are influenced by many aspects, such as fiber orientation, composition, reinforcement, adhesion, anisotropic, shape of inhomogeneities, manufacturing process, etc. Doing the tests on standard examples and assessing mechanical properties is the aspect of utmost importance in planning the applications of composite materials. The failure mechanisms and micromechanics of composites are very intricate compared to those of isotropic materials. Failure mechanisms and applicable theory are considered for the radomes, depending on the percentage, reinforcement, and composition content.

Here, ANSYS WORKBENCH is used to carry out the finite element analysis (FEA) of a submarine radome. A geometrical prototype of the radome is produced according to the radome sketch. Appropriate components are chosen, and ideal size of mesh is created. Material properties are assigned according to the specifications. Different loads and boundary conditions are applied to finish the preprocessing stage. The results are compared with the design requirements. The fundamental goal of this project is to create a composite radome that ensures the electronic hardware is protected from heavy water pressure and translucent to electromagnetic waves. Geometrically, the radome is similar to the cylinder which is covered by the dome which is hemispherical in shape, and it is bolted on the submarine from the lower side with help of a circular plate. GFRP and CFRP sandwiched structures are used for construction. The upper and lower laminates are made up from GFRP and CFRP face sheets, and six different materials are used as core materials. Combinations of different fabrics and resins are used and are compared with the design requirements.

Submarines are widely used all over the world for military purposes. In this article, we will use different combinations of materials and assess which one is more suitable for our application. Because this radome is a submarine radome, we must consider the increasing pressure while going down under water. It is imperative that the dielectric constant of the material is low. Deflections are reduced by the low-dielectric-constant material, and this further causes the insertion loses and impact of radiation pattern to be minimized.

## 2. Composite Failure Criteria

The safe and efficient use of materials is the biggest requirement for the effective design of a structure. Maximum stress criterion is a common methodology for using the limit theory. Each ply has the principal stresses, which are compared by their corresponding values of strength—*X_t_*, *Y_t_*, *X_c_*, *Y_c_*, and *S*—where *X_c_* and *X_t_* are compressive and longitudinal tensile strength, respectively; *Y_c_* and *Y_t_* are the same in the transverse direction; and *S* is the ultimate in-plane shear strength [16]. This criterion shows that whenever one of the principal stress components becomes greater than its corresponding strength, the failure will be predicted. Tensile stresses cause different types of failure when they are applied to any lamina. These failure types include transverse matrix cracking in the plane of the lamina, fiber breakage, and interfiber shear failure of the matrix. Fiber buckling will be produced if there are compressive stresses, and this buckling leads to failure along the fiber direction of lamina and matrix crushing, causing the composite matrix failure. This introduces the term ‘failure index’ defined as follows [17]:

(1) $$I_{F}=\{\begin{array}{c} \sigma_{11}/X_{t}\\ \sigma_{22}/Y_{t}\\ \tau_{12}/S \end{array}\;\mathrm{if}\;\sigma_{11}>0\;\mathrm{or}\;-\sigma_{11}/X_{c}\;\mathrm{if}\;\sigma_{11}<0\;\mathrm{if}\;\sigma_{11}>0\;\mathrm{or}\;-\sigma_{11}/Y_{c}\;\mathrm{if}\;\sigma_{22}<0$$

The most comprehensive criterion is the Tsai-Wu failure criterion [18], as it differentiates between the tensile and compressive strength of a lamina and can be expanded and written in the following form:

(2) $$I_{F}=F_{11}\sigma_{11}^{2}+F_{22}\sigma_{22}^{2}+F_{66}\tau_{12}^{2}+F_{1}\sigma_{11}+F_{2}\sigma_{22}+2F_{12}\sigma_{11}\sigma_{22}$$

where the parameters $\sigma_{11}$ and $\sigma_{22}$ are the stresses in the longitudinal and transverse fiber directions, respectively. In-plane shear stress is denoted by $\tau_{12}$. These are acquired from the following relationship:

(3) $$\left(\begin{array}{c} \sigma_{11}\\ \sigma_{22}\\ \tau_{12} \end{array}\right)=\left[\begin{array}{ccc} Q_{11} & Q_{12} & 0\\ Q_{12} & Q_{22} & 0\\ 0 & 0 & Q_{66} \end{array}\right]\left(\begin{array}{c} \varepsilon_{11}\\ \varepsilon_{22}\\ \gamma_{12} \end{array}\right)$$

The coefficients of Equation (2) are defined as $F_{11}=\frac{1}{X_{t}X_{c}}$, $F_{22}=\frac{1}{Y_{t}Y_{c}}$, $F_{66}=\frac{1}{S^{2}}$, $F_{1}=\frac{1}{X_{t}}-\frac{1}{X_{c}}$, $F_{2}=\frac{1}{Y_{t}}-\frac{1}{Y_{c}}$, $F_{12}=-\frac{1}{2}\sqrt{F_{11}F_{22}}$.

The failure takes place when the value of $I_{F}$ reaches or exceeds the value of 1.

Another criterion used to see failure is the Tsai-Hill criterion [19]. This theory is centered on the distortion energy failure theory of the von Mises distortional energy yield criterion for isotropic materials as applied to anisotropic materials. Distortion energy is actually a part of the total strain energy in a body. There are two parts of strain energy in a body; the one which is due to the change in shape is called distortion energy, and the other is due to volume and is called the dilation energy. There is a condition that a material will fail if the distortion energy is bigger than the failure distortion energy of the material. Hill implemented the von Mises distortional energy yield criterion to anisotropic materials. Then, Tsai amended it for the unidirectional lamina. He proposed on the basis of distortion energy theory that a lamina has failed if $\left(G_{2}+G_{3}\right)\sigma_{1}^{2}+\left(G_{1}+G_{3}\right)\sigma_{2}^{2}+\left(G_{1}+G_{2}\right)\sigma_{3}^{2}-2G_{3}\sigma_{1}\sigma_{2}-2G_{2}\sigma_{1}\sigma_{3}-2G_{1}\sigma_{2}\sigma_{3}+2G_{4}\tau_{23}^{2}+2G_{5}\tau_{13}^{2}+2G_{6}\tau_{12}^{2}<1$ is violated. The components $G_{1}$,$\;G_{2}$, $G_{3}$, $G_{4}$, $G_{5}$, and $G_{6}$ of the strength criterion can be calculated by the following equations and depend on failure strength.

(4) $$G_{1}=\frac{1}{2}\left(\frac{2}{{\left[{\left(\sigma_{2}^{T}\right)}_{ult}\right]}^{2}}-\frac{1}{{\left[{\left(\sigma_{1}^{T}\right)}_{ult}\right]}^{2}}\right)$$

(5) $$G_{2}=\frac{1}{2}\left(\frac{1}{{\left[{\left(\sigma_{1}^{T}\right)}_{ult}\right]}^{2}}\right)$$

(6) $$G_{3}=\frac{1}{2}\left(\frac{1}{{\left[{\left(\sigma_{1}^{T}\right)}_{ult}\right]}^{2}}\right)$$

(7) $$G_{6}=\frac{1}{2}\left(\frac{1}{{\left[{\left(\tau_{12}\right)}_{ult}\right]}^{2}}\right)$$

where $\sigma_{1}$, $\sigma_{2}$, and $\sigma_{3}$ are the stresses in direction 1, 2, and 3, respectively; $\tau_{12}$, $\tau_{13}$, and $\tau_{23}$ are the shear stresses in the respective plane; ${\left(\sigma_{1}^{T}\right)}_{ult}$ and ${\left(\sigma_{2}^{T}\right)}_{ult}$ are the ultimate tensile stresses in direction 1 and 2; and ${\left(\tau_{12}\right)}_{ult}$ is the ultimate shear stress in the respective plane mentioned in the subscript.

By putting the values and assumptions in an equation, it becomes

(8) $${\left[\frac{\sigma_{1}}{{\left(\sigma_{1}^{T}\right)}_{ult}}\right]}^{2}-\left[\frac{\sigma_{1}\sigma_{2}}{{\left(\sigma_{2}^{T}\right)}^{2}{}_{ult}}\right]+{\left[\frac{\sigma_{2}}{{\left(\sigma_{2}^{T}\right)}_{ult}}\right]}^{2}+{\left[\frac{\tau_{12}}{{\left(\tau_{12}\right)}_{ult}}\right]}^{2}<1$$

Given the comprehensive stresses in the lamina, we can find the local stresses and apply the above failure theory to define whether there is failure in the lamina or not.

### 2.1. Material Constraints

In order to confirm the stability of structure, the strength and failure constraints enforced on the radome must be gratified. Numerous strength and failure criteria are used as behavior constraints. The factor of safety for the Tsai-Wu and Tsai-Hill failure criteria are called material constraints for the composite material. Both the criteria must be greater than 1 for the composite material to remain safe. The von Mises stress must be smaller than the yield stress of the material.

$$SF_{TW}\geq 1$$

$$SF_{TH}\geq 1$$

$$\sigma_{YS}\geq \sigma_{VM}$$

### 2.2. Instability Constraint (Buckling Constraint)

The buckling factor *λ* is used as an instability constraint. This is actually the ratio of pressure applied to the critical buckling pressure, and it also must be greater than 1 to ensure that the material is safe.

λ≥1

## 3. Materials and Methods

We have used carbon/epoxy and glass/epoxy materials in the skin of sandwich structure. The mechanical properties of these materials are given below in Table 1.

Six different cores were used in the radome sandwich structure. The properties of cores are listed below in the Table 2.

### 3.1. Submarine Radome

Because our model is a submarine radome and it is submerged in water, water directly applies pressure normal to the submarine surface. This pressure is calculated by the following formula:

(9) P=ρgh

where ρ is the density of the fluid, g is gravitational acceleration, and h is the height of the fluid. The value of ρ for the sea water at standard temperature is 997.047 $\mathrm{kg}/m^{3}$.

Assuming radome depth in water is 500 m,

$$P=\left(997.047\right)\left(9.81\right)\left(500\right)=4890515.54{\;\mathrm{Nm}}^{2}=48.905\;bar=4.90\;\mathrm{MPa}$$

The maximum depth at which a submarine can operate is called the maximum operating depth. Below this maximum operating depth, the material will fail due to the pressure of water. So, we have to design our radome accordingly. The dimensions of the radome are taken from [27] and mentioned in Table 3.

We have to assume some conditions for our model:

- The radome is fixed in all degrees of freedom at the lower end.
- The *y*-axis direction is used as a reference direction for fiber orientation.
- Water pressure is acting normal to the periphery of the radome.
- The orientations for the fibers of FRP are taken as [45/−45] and [0/90] degrees.

### 3.2. Method of Finite Element Model of the Submarine Radome

**Figure 1:**
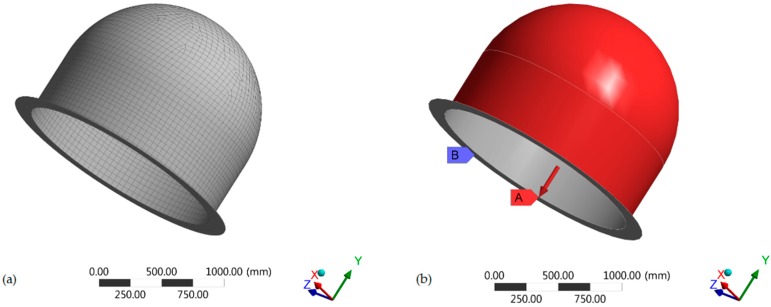
(**a**) Meshing of the radome model; (**b**) boundary and loading conditions of the radome.

The finite element model of the submarine is developed using shell elements in ANSYS WORKBENCH. An element size of 50 mm is used for discretization of the finite element model. Initial model is developed in an ANSYS Composite PrePost (ACP) module. Then, this composite model is transferred to a static structure module for static analysis to determine the factor of safety for the composite failure. The data obtained from static module is then transferred to an eigenvalue buckling module to determine the value of the buckling factor, *λ.* Orthotropic material properties are used for composite skins, whereas isotropic material properties are used for polyvinyl chloride (PVC) and mulberry wood cores. The model is fixed at the lower end in all degrees of freedom. Pressure of 5.25 MPa is applied on the external surface. The meshed model, boundary conditions, and loading conditions are shown in Figure 1. The arrows A and B shows the loading and boundary conditions respectively. All the required parameters are parametrized in their respective module.

## 4. Results and Discussion

Table 4 shows the results obtained from the software ANSYS WORKBENCH. The left vertical column shows the materials which are used in the cores of our different radome samples. Both the layup types are shown in the laminate layup section. The minimum inner and outer thicknesses are mentioned in the table, which is the minimum value of thickness for which all conditions are satisfied and the material is safe. Then, the total deformation of the material is measured and mentioned in the deformation column. Three failure criteria, namely Tsai-Wu, Tsai-Hill, and eigenvalue buckling, are then mentioned in the failure criterion column. The value of von Mises stress for our desired thickness is also mentioned in a separate column. At the end, the total weight of the desired layup is mentioned. All values are for the minimum thickness for which all the parameters are safe. The values beyond these are mentioned in graphs in the sensitivity analysis section.

### 4.1. Layup [45/−45]

Six different sorts of core materials are used for the optimization of our radome structure. For the identical boundary conditions and loadings, as the submersible submarine radome, the cross-ply laminate [45/−45] is optimized for minimum weight and strength. The number of layers varied from 1 to 20. There are two conditions for the safe model. First of all, values of the Tsai-Wu ($SF_{TW}$) criterion, Tsai-Hill ($SF_{TH}$) criterion, and buckling strength factor λ must be greater than one. The second is that von Mises stress must be less than the tensile strength of the core material. The results obtained from PVC foam and five different wood cores are shown in Table 4.

For PVC foam, there is only one possible case for the CFRP skin and PVC foam core. The material is not safe in the case of the GFRP skin. The factor of safety for the Tsai-Wu ($SF_{TW}$) criterion, Tsai-Hill ($SF_{TH}$) criterion, and buckling strength factor λ is 2.916, 2.350, and 1.068, respectively. The laminate plies for inner and outer skins are ${\left[45_{11}/-45_{11}\right]}_{22}$, which means 22 plies are used in the inner skin and 22 are used in the outer skin with a PVC foam core of 55 mm thickness. This configuration makes the radome thicker and heavier. Its weight exceeds 571.9 kg. The value of von Mises stress is 2.402 MPa, which is low, but its tensile strength is 2.5 MPa. So, there is no significant difference in von Mises and tensile strength, explaining why its buckling strength factor is also low, which shows a lower factor of safety.

For the CFRP skin and bamboo wood core, the factor of safety for the Tsai-Wu ($SF_{TW}$) and Tsai-Hill ($SF_{TH}$) criteria is 1.437 and 1.135, respectively. The buckling strength factor λ in this case is 2.022. All these parameters are greater than 1, which means the material is safe for our required specifications. We have also checked the values of von Mises stress, which in this case, is 31.118 MPa, i.e., less than its yield strength, which is 128.53 MPa for bamboo wood. On the basis of these four parameters, it is concluded that all the combinations beyond ${\left[45_{4}/-45_{4}\right]}_{8}$, which means 8 layers of outer skin and 8 layers of inner skin with a core of 20 mm thickness, are safe for our required specifications. This is the minimum safe value from which the total deformation is recorded—6.195 mm—and the minimum possible weight of 315.74 kg for a submarine operating 500 m under the surface of water. In the case of the GFRP skin and bamboo wood core, the values of $SF_{TW}$, $SF_{TH}$, and λ are 1.334, 1.018, and 1.435, respectively. However, the value of von Mises stress is increased to 55.199 MPa for the same angle of laminated ply. The value of deformation and weight of the radome is also increased to 8.991 mm and 382.35 kg, respectively, for the same thickness of laminate. This shows that the CFRP skin is safer than the GFRP skin for the same cores.

For the CFRP skin and Paulownia wood core, the values of $SF_{TW}$, $SF_{TH}$, and λ are 1.263, 1.017, and 1.970, respectively, for the thickness of ${\left[45_{4}/-45_{4}\right]}_{8}$ for each ply. The value of von Mises stress is too low for bamboo and is equal to 18.961 MPa, although its tensile strength is equal to 49.10 MPa. However, for the same thickness, the weight is reduced to 240.32 kg, as compared to the bamboo wood core. So, the Paulownia wood core is comparatively better than the bamboo core. The value of deformation is also decreased to 6.776 mm. For the GFRP skin and Paulownia core, the safety and buckling factors are increased by 1.612, 1.232, and 2.143, respectively. Buckling is the main failure type in these types of cylindrical shapes under water. We can find the safe value of operating pressure by multiplying the desired pressure by the buckling factor. So, for this case, the material is safe under 11.25 MPa of external pressure. However, the thickness and weight are increased to 45 mm and 383.66 kg, respectively. The value of von Mises stress is increased to 24.549 MPa. We can see that there is no significant change in our constraints, but the weight increased too much for our desired structure.

Balsa wood is known as being the lightest wood in the world, with excellent properties. For the CFRP skin and balsa wood core, the values of $SF_{TW}$, $SF_{TH}$, and λ are 1.393, 1.163, and 3.162, respectively. The value of the buckling factor is high, but at the same time, its thickness and weight are increased to 45 mm and 294.28 kg, respectively. The laminate ply for inner and outer skin is ${\left[45_{5}/-45_{5}\right]}_{10}$ with a core of 25 mm thickness. The value of von Mises stress is 16.225 MPa, but its tensile strength is 23.50 MPa. Although its safe but there is no significant difference between von Mises stress and tensile yield strength. In the case of the GFRP skin and balsa wood core, the values of $SF_{TW}$, $SF_{TH}$, and λ are 1.416, 1.058, and 2.292, respectively. The value of the buckling factor is decreased as compared to the CFRP skin for the same thickness and equal number of plies. The total weight of the radome is decreased, but the value of von Mises stress is increased to 18.093 MPa. The value of deformation is also increased to 7.866 mm.

We have used two types of mulberry wood, namely mulberry and engineered mulberry. For the CFRP skin and pure mulberry, the values for $SF_{TW}$, $SF_{TH}$, and λ are 1.789, 1.273, and 4.279. Here, the buckling factor is increased, thus the radome made by this material is safe for 22.465 MPa of external pressure. The laminate plies for inner and outer skins are ${\left[45_{4}/-45_{4}\right]}_{8}$, which means 8 plies are used in the inner skin and 8 are used in the outer skin with a mulberry core of 20 mm thickness. The value of deformation is 4.635 mm, which is too low compared to the other cores. The value of von Mises stress is 51.855 MPa, which is low compared to the tensile strength of mulberry, which is equal to 605 MPa. The total weight for this configuration is 307.25 kg. For the GFRP skin and mulberry core, there is no significant change in the values of the constraints, being 1.641, 1.227, and 4.123, respectively, but the weight is increased abruptly to 467.33 kg. The value of thickness and von Mises stress is also increased to 45 mm and 56.238 MPa, respectively.

For the CFRP skin and engineered mulberry, the values for $SF_{TW}$, $SF_{TH}$, and λ are 1.780, 1.270, and 4.270. It is apparent that all the parameters have very similar values and thickness to the CFRP skin and mulberry wood core, but the weight of engineered mulberry is decreased to 292.56 kg. For the GFRP skin, the constraint values are also similar, but there is too much increase in weight and thickness.

### 4.2. Layup [0/90]

Next, the layup for the plies is changed to [0/90] for all the wood cores. For the same boundary conditions and loadings, as the submersible submarine radome, the cross-ply laminate [0/90] is optimized for minimum weight and strength.

For PVC, there is only one possible case for the CFRP skin and PVC foam core. The material is not safe in the case of the GFRP skin. The factor of safety for Tsai-Wu ($SF_{TW}$) criterion, Tsai-Hill ($SF_{TH}$) criterion, and buckling strength factor λ is 7.657, 5.295, and 1.205, respectively. Although all these factors are increased compared to the [45/−45] configuration, the thickness remained the same. The laminate plies for inner and outer skins are ${\left[45_{11}/-45_{11}\right]}_{22}$, which means 22 plies are used in the inner skin and 22 are used in the outer skin with a PVC foam core of 55 mm thickness. This configuration makes the radome thicker and heavier. Its weight exceeds 571.9 kg. The value of von Mises stress is 2.204 MPa, which is low, but its tensile strength is 2.5 MPa. So, there is no significant difference in the von Mises stress and tensile strength, explaining why its buckling strength factor is also low, which shows a lower factor of safety. The weight is same for both cases, but it is proved that the [0/90] version increased the values of the safety and buckling constraints.

For the CFRP skin and bamboo wood core, the factor of safety for the Tsai-Wu ($SF_{TW}$) and Tsai-Hill ($SF_{TH}$) criteria is 2.173 and 1.532, respectively. The buckling strength factor λ in this case is 1.052. Thickness is reduced to ${\left[0_{3}/90_{3}\right]}_{6}$ for each side, which means 6 layers of plies on each side with a 15 mm core of bamboo and total thickness of 27 mm. For the same conditions, there were eight layers for each side on this angle configuration ${\left[45_{4}/-45_{4}\right]}_{8}$ with a core thickness of 20 mm and total thickness of 36 mm. This proves that the values of constraints also depend on the angle of the plies. Values of deformation, von Mises stress, and weight are also decreased to 4.30 mm, 30.905 MPa, and 236.81 kg, respectively. In the case of GFRP, the values of $SF_{TW}$, $SF_{TH}$, and λ are 1.401, 1.264, and 1.321, respectively. Thickness of radome is increased to 36 mm, due to which the overall weight is also increased to 382.35 kg. Deformation and von Mises stress is also increased to 6.758 mm and 46.281 MPa, respectively.

For the Paulownia wood core and CFRP skin, the values of $SF_{TW}$, $SF_{TH}$, and λ are 2.121, 1.490, and 1.023, respectively. The thickness of core is also decreased to 27 mm as compared to the ${\left[45_{4}/-45_{4}\right]}_{8}$ configuration. The value of von Mises stress is 18.543 MPa, which is almost same for the [45/−45] configuration. However, the weight is decreased to 180.24 kg due to decrease in the thickness. For the GFRP skin and Paulownia wood core, the values of $SF_{TW}$, $SF_{TH},$ and λ are 1.331, 1.186, and 1.273, respectively. The value of deformation is increased to 7.074 mm, which was 4.393 mm in the case of the CFRP skin configuration. The values of von Mises and weight are also increased to 26.330 MPa and 306.93 kg, respectively, which were 18.543 MPa and 180.24 kg, respectively, in the case of the CFRP skin. So, the [0/90] configuration of the radome with the CFRP skin is the best configuration for the Paulownia wood core.

For the balsa wood core with the CFRP skin, the values of $SF_{TW}$, $SF_{TH}$, and λ are 2.047, 1.425, and 1.077, respectively. The buckling factor is decreased, as it was 3.162 for the [45/−45] configuration. The smallest value of thickness, 27 mm, is obtained from all configurations of balsa. The value of deformation in this case is 4.527 mm. No significant change occurred in the von Mises stress value, and the value of 17.632 MPa is obtained for this case. The weight obtained in this configuration is lowest of all and was recorded as 176.57 kg. In the case of the GFRP skin, the values of the respective constraints are 1.255, 1.094, and 1.332. Although there is no significant change in the values of buckling and safety factors, the weight increases substantially to 302.033 kg. Deformation is doubled compared to the CFRP skin. The von Mises value jumps to 20.332 MPa.

For pure mulberry, the values for $SF_{TW}$, $SF_{TH}$, and λ are 1.917, 1.561, and 1.739. All the values are perfect, with minimum deformation of 3.773 mm and minimum weight of 230.44 kg. The value of von Mises is recorded as 48.249 MPa for the minimum thickness of 27 mm. This is the best possible configuration for the mulberry core. For the GFRP skins and mulberry wood core, the values of the safety and buckling constraints are smaller than the CFRP skin and are obtained as 1.336, 1.131, and 2.131, respectively. Overall thickness and weight are increased to 36 mm and 373.86 kg, respectively. Deformation and von Mises values are also increased to 5.112 mm and 64.443 MPa, respectively.

For the CFRP skin and engineered mulberry wood, the values for $SF_{TW}$, $SF_{TH}$, and λ are 1.919, 1.559, and 1.735, respectively. This is the best combination among the engineered mulberry wood. The thickness and weight are lowest from all combinations and are equal to 27 mm and 219.42 kg, respectively. The value of von Mises stress is also decreased and is equal to 47.743 MPa. Deformation is 3.782 mm, which is almost equal to the mulberry wood with the same configuration. In the case of the GFRP skin, the values of the safety and buckling constraints are 1.135, 1.130, and 2.126, respectively. Thickness is increased to 36 mm, again due to which its overall weight is increased to 359.17 kg. Values of von Mises stress and deformation are also increased to 63.882 MPa and 5.133 mm, respectively.

### 4.3. Best Radome Configuration

By analyzing all the results obtained from the simulations, we found the best two possible configurations of wood cores, i.e., balsa and engineered mulberry. We will discuss different parameters of both, and on the basis of that knowledge, we will then select one for our desired application. The thickness of both cores is 27 mm. Safety factor constraints are also almost equal. So, there are three main things to discuss; i.e., the buckling factor, weight, and tensile strength. The buckling factor of engineered mulberry is higher than balsa, but the weight of balsa is less than engineered mulberry. Weight of the balsa core is 176.57 kg, whereas for engineered mulberry, it is 219.42 kg. Regarding the value of tensile strength, engineered mulberry has the strength of 646 MPa, which is very high compared to balsa, which is only 23.50 MPa. Further, this engineered mulberry wood is made by processing the small branches of mulberry wood, which are cheap and easily available. So, we have selected the engineered mulberry wood core for further analysis. The values of different parameters for this wood are given in the following figures which are taken from ANSYS WORKBENCH.

**Figure 2:**
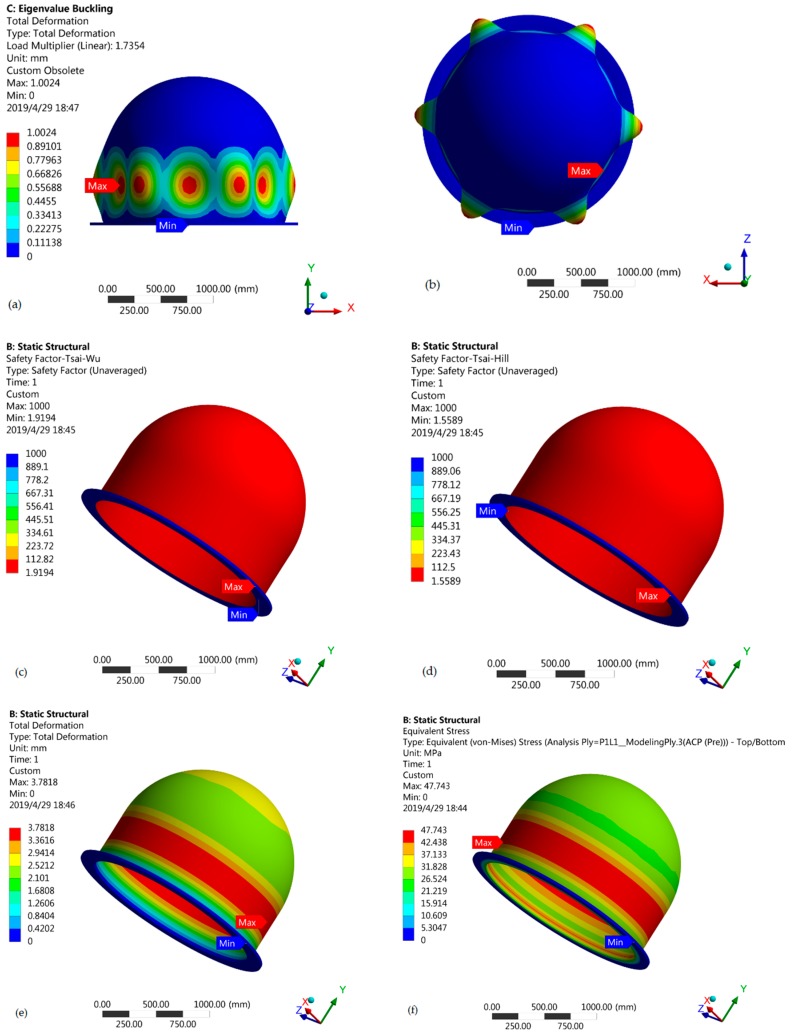

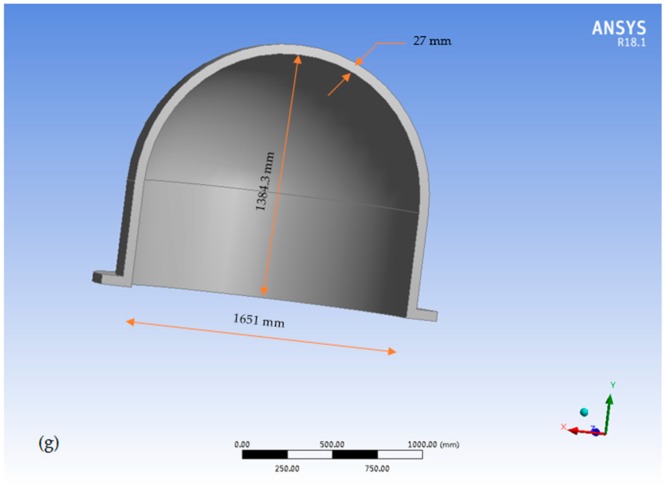
(**a**) Eigenvalue of buckling; (**b**) top view showing buckling; (**c**) values of the Tsai-Wu safety factor; (**d**) Values of the Tsai-Hill safety factor; (**e**) values of total deformation; (**f**) values of von Mises stress; (**g**) dimensions of the best radome configuration.

Figure 2a shows the final values of eigenvalue buckling for the engineered mulberry wood core. Buckling starts from the sides. Red color shows the place where the value of the buckling factor is maximum, i.e., 1.004. There is no buckling on the top and lower places of the radome. Figure 2b shows the top view, from which we can see the exact shape of the radome after buckling. Figure 2c,d shows the values of the Tsai-Wu and Tsai-Hill safety factors, respectively. For the Tsai-Wu factor, the minimum value of 1.9194 is shown by red color all over the radome. For the Tsai-Hill factor, the minimum value is 1.5589. In both cases, the radome base is the safest position, with the maximum value of the safety factor. Figure 2e shows the value of total deformation. It is clear from the figure that the maximum value of 3.7818 mm is right in the center, where there is the maximum value of buckling. Deformation takes place from that position where the buckling starts and it reduces heading towards the base. The minimum value is present on the base. We can see the same trend for von Mises stress in Figure 2f, as it has also the maximum value of 47.743 MPa at the center. The value of von Mises stress on the top of radome structure is almost half that of the center.

## 5. Sensitivity Analysis

**Figure 3:**
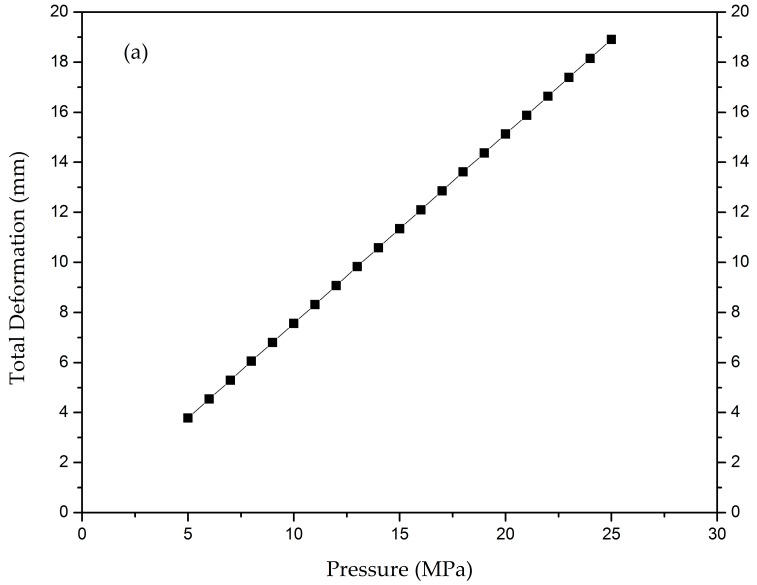

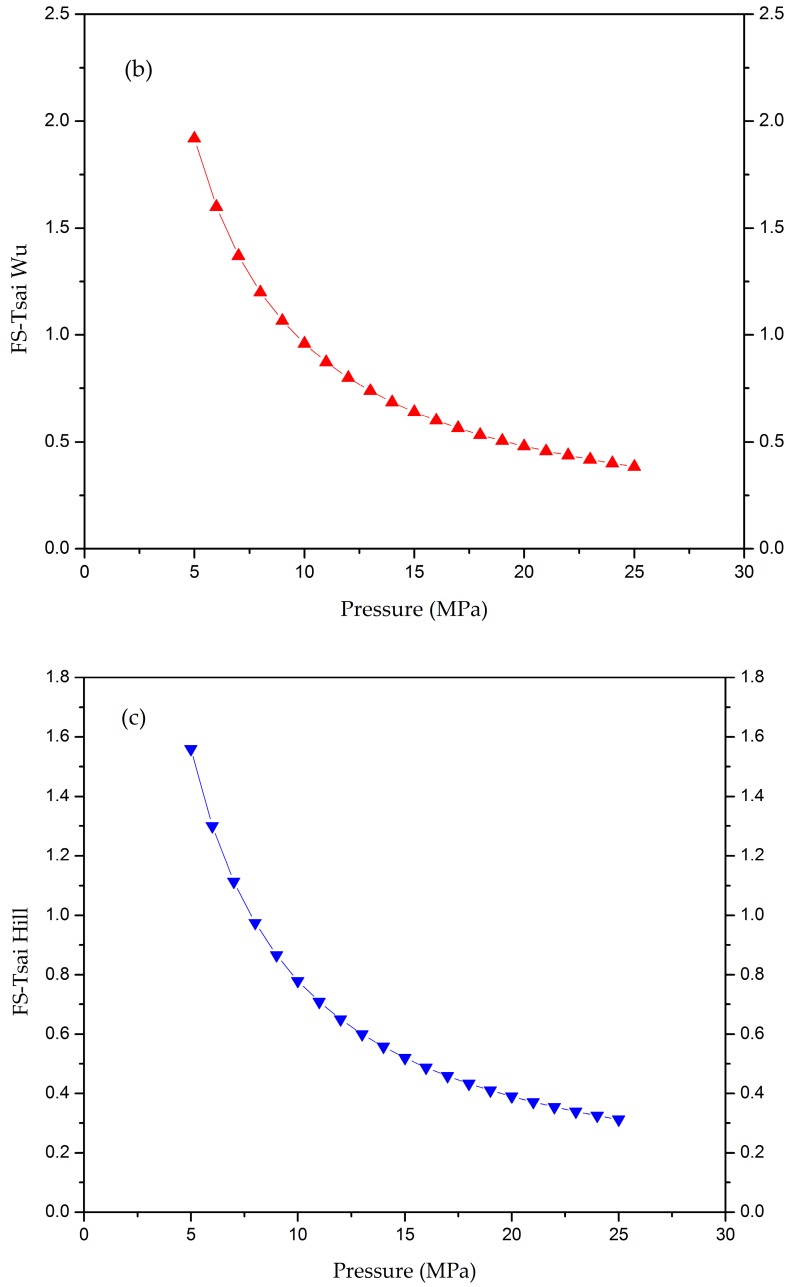

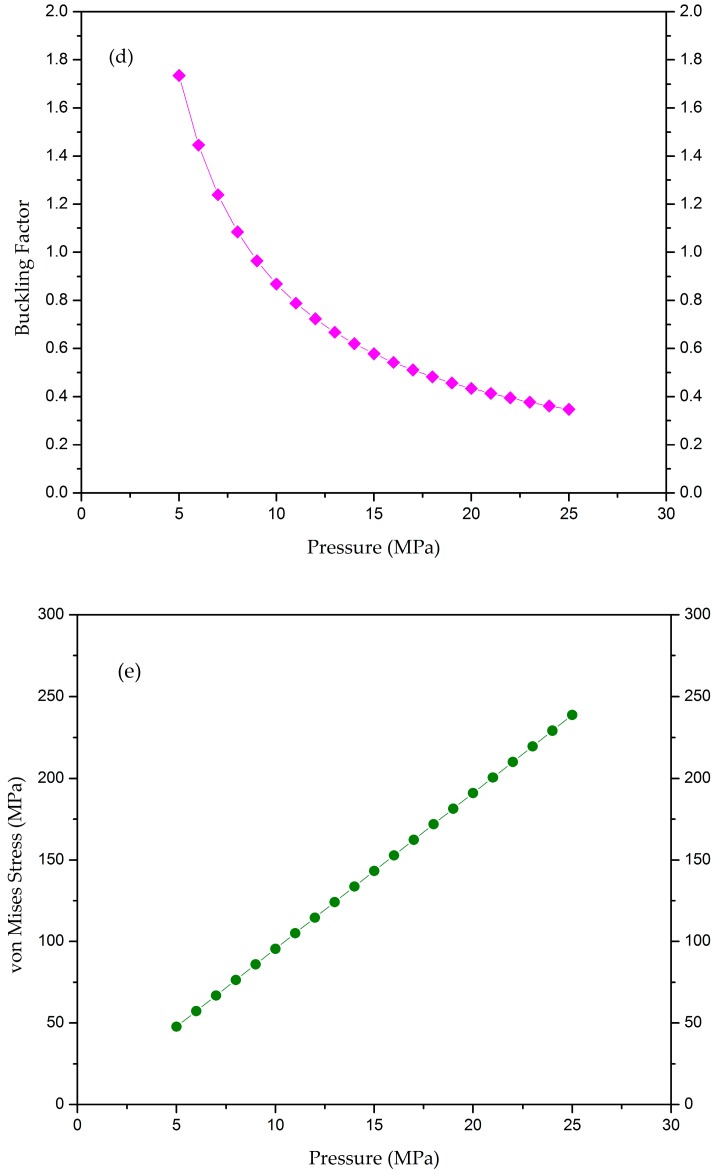
(**a**) Graph of total deformation vs. pressure; (**b**) graph of Tsai-Wu safety factor vs. pressure; (**c**) graph of Tsai-Hill safety factor vs. pressure; (**d**) graph of eigenvalue buckling vs. pressure; (**e**) graph of von Mises stress vs. pressure.

A sensitivity analysis is done to comprehensively assess the effects of the design variables on the structural strength of the submarine radome under hydrostatic pressure. The layup ${\left[0_{3}/90_{3}\right]}_{6}$ for the engineered mulberry core is selected to assess sensitivity. The first layer shows the orientation angle (0°), and the second layer shows the orientation angle (90°) with six layers inside and six outside the core. Results attained are presented and summarized in Figure 3a–e, which are discussed below.

### 5.1. Effect of Pressure and Design Variables on Total Deformation

We varied the pressure from 5 MPa to 25 MPa on our most suitable configuration, i.e., the CFRP engineered mulberry core submarine radome. Deformation was reported to be from 3.78 mm to 18.91 mm. The material was safe up until 7 MPa, as the safety constraints and buckling factor were greater than 1. It is clear from Figure 3a that at 7 MPa, the value of deformation is 5.295 mm. From the graph, it can be seen that when the external pressure is increasing, the value of total deformation is also increasing, and it reached 18.91 mm with 25 MPa pressure. It can also be seen that on average, 0.80 mm of deformation occurred with an increase in pressure of 1 MPa.

### 5.2. Effect of Pressure and Design Variables on Safety Factor Constraints

Tsai-Wu and Tsai-Hill safety factors are used as material failure constraints. We analyzed the values from our desired layup. Pressure was varied from 5 to 25 MPa, and the values of Tsai-Wu and Tsai-Hill safety factors were observed. When the pressure was 5 MPa, the value of $FS_{TW}$ was 1.919, and at the maximum pressure of 25 MPa, its value dropped to 0.3838. For safe material, its value must be one or greater than one. At the pressure of 9 MPa, the value is 1.066, as shown in the Figure 3b; this means that according to Tsai-Wu theory, this radome is safe at pressures up to 9 MPa. After 9 MPa, its values start to become less than 1. There are three zones in the graph, as shown in Figure 3b. In the first zone, the value drops more quickly; the second zone shows that the value drops by decrements of 0.1; and the third zone shows the trend that the value gradually becomes parallel to the axis.

In the case of $FS_{TH}$, the value drops more quickly to zero. The value starts from 1.559 with the pressure of 5 MPa, and at 7 MPa, its value becomes 1.113. After that, the value becomes less than zero. So, we can say that according to the Tsai-Hill failure criterion, this radome is safe for up to 7 MPa hydrostatic pressure, as shown in the Figure 3c. If we compare the values with the Tsai-Wu criterion, all the values of the $FS_{TH}$ criterion are less than $FS_{TW}$ at all pressure points. At the end of the graph, both criteria show very similar behavior.

### 5.3. Effect of Pressure and Design Variables on the Buckling Factor

Buckling is the main failure usually experienced in these types of submersible shapes. For example, Blachut et al. described a series of experimental and numerical studies on the buckling enactment of hemispherical [28,29] and torispherical [30] domes subjected to external pressure. These studies explained that the geometry of a radome intensely affects its service performance and buckling resistance. So, the buckling factor λ is used to analyze the buckling in the radome. This factor must be greater than 1 if the material is to remain safe from buckling. The value of the buckling factor starts at 1.735 at 5 MPa hydrostatic pressure. The radome is safe from buckling until 8 MPa pressure, because at this pressure, its value is 1.084, as shown in Figure 3d. After that, the values become smaller than zero and buckling starts. The buckling trend is almost the same as that of the Tsai-Wu failure criterion.

### 5.4. Effect of Pressure and Design Variables on von Mises Stress

The last important parameter to analyze for the material is the value of von Mises stress. For safe material, the value of von Mises stress must be less than the value of its tensile strength. For our core material, the value of tensile strength is 646 MPa. All the values are below 646 MPa. The value starts at 47.743 MPa at 5 MPa hydrostatic pressure. The value increases by 10 MPa for every rise of 1 MPa in pressure, as shown in Figure 3e. At 25 MPa, its value is 238.714 MPa, which is still in the safe range.

## 6. Conclusions

Low-dielectric-constant materials are used in the core of sandwich structures of submarine radomes, so that the electromagnetic waves from radar do not interfere with the radome material. Wood and foam have low dielectric constants as compared with other materials. We used six different core materials of wood and foam to numerically investigate the behavior and failure of a radome at an underwater depth of 500 m. Failure criterion theories were applied to calculate safety constraints; i.e., the Tsai-Wu, Tsai-Hill, and buckling factors. We took the configurations in which the values of these factors were greater than 1 as the safe model. Total deformation and von Mises stresses were also calculated, and those configurations were selected in which the values were smaller than tensile strength of the core materials. Two types of layups [45/−45] and [0/90] were used with GFRP and CFRP face sheets. It is shown in the article that layup angle has a great effect on the strength of the material. The best radome configuration, with an engineered mulberry wood core, was selected by comparing the parameters with different cores. The experimental validation of this study will be performed in future research on submarine radomes.

## Figures and Tables

**Table 1:** Mechanical properties of glass/epoxy and carbon/epoxy materials [20].

Mechanical Properties	Glass/Epoxy	Carbon/Epoxy
Elastic Modulus		
$E_{1}$	45,000 (MPa)	1.21 × 10^5^ (MPa)
$E_{2}$	10,000 (MPa)	8600 (MPa)
$E_{3}$	10,000 (MPa)	8600 (MPa)
Shear Modulus		
$G_{12}$	5000 (MPa)	4700 (MPa)
$G_{13}$	5000 (MPa)	4700 (MPa)
$G_{23}$	3846.2 (MPa)	3100 (MPa)
Density	2000 ($\mathrm{kg}/m^{3}$)	1490 ($\mathrm{kg}/m^{3}$)
Poisson Ratio		
${\mathrm{Nu}}_{12}$	0.3	0.27
${\mathrm{Nu}}_{13}$	0.3	0.27
${\mathrm{Nu}}_{23}$	0.4	0.40
Tensile Strength		
${\mathrm{TS}}_{1}$	1100 (MPa)	2231 (MPa)
${\mathrm{TS}}_{2}$	35 (MPa)	29 (MPa)
${\mathrm{TS}}_{3}$	35 (MPa)	29 (MPa)

**Table 2:** Mechanical properties of wood core materials [21,22,23,24,25,26].

Mechanical Properties	Paulownia Wood	Bamboo	Mulberry Wood/Engineered Mulberry	Balsa Wood	PVC Foam
Elastic Modulus					
$E_{1}$	4.32 × 10^9^ (Pa)	1.00 × 10^10^ (Pa)	9320 (MPa)	200 (MPa)	
$E_{2}$	1.47 × 10^9^ (Pa)	2.50 × 10^9^ (Pa)	9200 (MPa)	4320 (MPa)	102 (MPa)
$E_{3}$	1.47 × 10^9^ (Pa)	2.50 × 10^9^ (Pa)		200 (MPa)	
Shear Modulus					
$G_{12}$	2.94 × 10^8^ (Pa)	2.75 × 10^8^ (Pa)	3.5 × 10^9^ (Pa)	354 (MPa)	
$G_{13}$	2.09 × 10^8^ (Pa)	2.75 × 10^8^ (Pa)	3.48 × 10^9^ (Pa)	309 (MPa)	39.2 (MPa)
$G_{23}$	2.94 × 10^8^ (Pa)	2.75 × 10^8^ (Pa)		64 (MPa)	
Density ($\mathrm{kg}/m^{3}$)	280	742	690/600	250	80
Poisson Ratio					
$Nu_{12}$	0.23	0.31	0.32	0.23	
$Nu_{13}$	0.23	0.31		0.49	0.3
$Nu_{23}$	0.23	0.31		0.66	
Tensile Strength (MPa)	49.10	128.53	605	23.50	2.5
			646		

PVC-polyvinyl chloride.

**Table 3:** Radome dimensions.

Parameter	Value in mm
Diameter	1651
Height	1384.3

**Table 4:** Results obtained from ANSYS WORKBENCH.

Material			Laminate Layup		Thickness				Deformation	Failure Criterion				Total Weight
Core	Inner Skin	Outer Skin	Inner Skin	Outer Skin	Inner Skin (mm)	Outer Skin (mm)	Core (mm)	Total	mm	Tsai-Wu	Tsai-Hill	Buckling Factor	von Mises Stress MPa	kg
PVC	CFRP	CFRP	${\left[45_{11}/-45_{11}\right]}_{22}$	${\left[45_{11}/-45_{11}\right]}_{22}$	22	22	55	99	2.960	2.916	2.350	1.068	2.402	571.09
			${\left[0_{11}/90_{11}\right]}_{22}$	${\left[0_{11}/90_{11}\right]}_{22}$	22	22	55	99	1.224	7.657	5.295	1.205	2.204	571.09
PVC	GFRP	GFRP	${\left[45_{11}/45_{11}\right]}_{22}$	–	–	–	–	–	–	–	–	–	–	–
Bamboo	CFRP	CFRP	${\left[45_{4}/-45_{4}\right]}_{8}$	${\left[45_{4}/-45_{4}\right]}_{8}$	8	8	20	36	6.195	1.437	1.135	2.022	31.118	315.74
			${\left[0_{3}/90_{3}\right]}_{6}$	${\left[0_{3}/45_{3}\right]}_{6}$	6	6	15	27	4.300	2.173	1.532	1.052	30.905	236.81
Bamboo	GFRP	GFRP	${\left[45_{4}/-45_{4}\right]}_{8}$	${\left[45_{4}/-45_{4}\right]}_{8}$	8	8	20	36	8.991	1.334	1.018	1.435	55.199	382.35
			${\left[0_{4}/90_{4}\right]}_{8}$	${\left[0_{4}/90_{4}\right]}_{8}$	8	8	20	36	6.758	1.401	1.264	1.321	46.281	382.35
Paulownia	CFRP	CFRP	${\left[45_{4}/-45_{4}\right]}_{8}$	${\left[45_{4}/-45_{4}\right]}_{8}$	8	8	20	36	6.776	1.263	1.017	1.970	18.961	240.32
			${\left[0_{3}/90_{3}\right]}_{6}$	${\left[0_{3}/90_{3}\right]}_{6}$	6	6	15	27	4.393	2.121	1.490	1.023	18.543	180.24
Paulownia	GFRP	GFRP	${\left[45_{5}/-45_{5}\right]}_{10}$	${\left[45_{5}/-45_{5}\right]}_{10}$	10	10	25	45	7.415	1.612	1.232	2.143	24.549	383.66
			${\left[0_{4}/90_{4}\right]}_{8}$	${\left[0_{4}/90_{4}\right]}_{8}$	8	8	20	36	7.074	1.331	1.186	1.273	26.330	306.93
Balsa	CFRP	CFRP	${\left[45_{5}/-45_{5}\right]}_{10}$	${\left[45_{5}/-45_{5}\right]}_{10}$	10	10	25	45	5.716	1.393	1.163	3.162	16.225	294.28
			${\left[0_{3}/90_{3}\right]}_{6}$	${\left[0_{3}/90_{3}\right]}_{6}$	6	6	15	27	4.527	2.047	1.425	1.077	17.632	176.57
Balsa	GFRP	GFRP	${\left[45_{5}/-45_{5}\right]}_{10}$	${\left[45_{5}/-45_{5}\right]}_{10}$	10	10	25	45	7.866	1.416	1.058	2.292	18.093	377.54
			${\left[0_{4}/90_{4}\right]}_{8}$	${\left[0_{4}/90_{4}\right]}_{8}$	8	8	20	36	7.455	1.255	1.094	1.332	20.332	302.03
Mulberry	CFRP	CFRP	${\left[45_{4}/-45_{4}\right]}_{8}$	${\left[45_{4}/-45_{4}\right]}_{8}$	8	8	20	36	4.635	1.789	1.273	4.279	51.855	307.25
			${\left[0_{3}/90_{3}\right]}_{6}$	${\left[0_{3}/90_{3}\right]}_{6}$	6	6	15	27	3.773	1.917	1.561	1.739	48.249	230.44
Mulberry	GFRP	GFRP	${\left[45_{5}/-45_{5}\right]}_{10}$	${\left[45_{5}/-45_{5}\right]}_{10}$	10	10	25	45	4.818	1.641	1.227	4.123	56.238	467.33
			${\left[0_{4}/90_{4}\right]}_{8}$	${\left[0_{4}/90_{4}\right]}_{8}$	8	8	20	36	5.112	1.136	1.131	2.131	64.443	373.86
Eng-Mul	CFRP	CFRP	${\left[45_{4}/-45_{4}\right]}_{8}$	${\left[45_{4}/-45_{4}\right]}_{8}$	8	8	20	36	4.655	1.780	1.270	4.270	51.417	292.56
			${\left[0_{3}/90_{3}\right]}_{6}$	${\left[0_{3}/90_{3}\right]}_{6}$	6	6	15	27	3.782	1.919	1.559	1.735	47.743	219.42
Eng-Mul	GFRP	GFRP	${\left[45_{5}/-45_{5}\right]}_{10}$	${\left[45_{5}/-45_{5}\right]}_{10}$	10	10	25	45	4.841	1.635	1.222	4.113	55.767	448.96
			${\left[0_{4}/90_{4}\right]}_{8}$	${\left[0_{4}/90_{4}\right]}_{8}$	8	8	20	36	5.133	1.135	1.130	2.126	63.882	359.17

CFRP-carbon fiber reinforced polymer, GFRP-glass fiber reinforced polymer, Eng-Mul-Engineered Mulberry.
